# A Case Report of Recombinant Tissue Plasminogen Activator Use in a SPAN-100-Positive Geriatric Patient with Thrombocytopenia

**DOI:** 10.7759/cureus.1933

**Published:** 2017-12-11

**Authors:** Ilya Bragin, Justine M Chen

**Affiliations:** 1 Neurology, St. Lukes University Health Network; 2 Neurology, Columbia University

**Keywords:** tpa, stroke, thrombocytopenia, span-100, contraindications to tpa, thrombolytic, ischemic stroke, alteplase, cerebrovascular accident, cva

## Abstract

Thrombocytopenia (platelet count: < 100,000/mm^3^) is considered a contraindication in the use of intravenous thrombolysis for acute ischemic stroke. Little literature exists regarding tissue plasminogen activator (tPA) usage in thrombocytopenic patients, especially in older patients. Age and stroke severity are major prognostic indicators of the risk of hemorrhagic transformation. The Stroke Prognostication using Age and NIH Stroke Scale (SPAN) index estimates a patient’s risk of intracerebral hemorrhage (ICH) and clinical response to thrombolysis by combining age in years with the National Institutes of Health Stroke Scale (NIHSS) scores. If the total numeric sum is 100 or more, these individuals are considered SPAN-100-positive, while those with a sum less than 100 are considered SPAN-100-negative patients. SPAN-100-positive patients are found to have a greater risk of ICH and poorer long-term outcomes than SPAN-100-negative patients both with and without thrombolysis treatment. SPAN-100-positive patients are found to have a greater risk of ICH and poorer long-term outcomes than SPAN-100-negative patients both with and without thrombolysis treatment. Nonetheless, SPAN-100-positive patients treated with tPA have a reduced relative likelihood of severe disability or death than SPAN-100-positive patients not treated with tPA. We report a case of a SPAN-100-positive, 90-year-old community-dwelling patient who presented with an acute ischemic stroke, an NIHSS score of 14 with near complete left-sided plegia, and a platelet count of 85,000/mm^3^. Our patient was at increased risk of ICH and poor outcome regardless of tPA administration. However, due to the patient’s high functional capacity prior to hospitalization and probable severe morbidity with poor recovery potential at his age, he was treated with tPA and showed a rapid improvement in neurological symptoms with no thrombolytic-associated morbidity. Thrombolytic therapy requires a case-by-case approach. Taking into account the patient’s baseline and recovery potential is critical. Even absolute and relative contraindications, as they stand now, may need reconsideration, particularly those with little empiric evidence. More research is indicated to establish if thrombocytopenia should be reclassified as a relative rather than absolute contraindication to tPA.

## Introduction

According to the 2013 American Heart Association (AHA)/American Stroke Association (ASA) Guidelines and the previous manufacturer label for alteplase, the tissue plasminogen activator (tPA) licensed for use in acute ischemic stroke, thrombocytopenia (platelet count < 100,000/mm^3^) is a contraindication for the use of tPA in patients with acute ischemic stroke [1]. Since 2015, however, thrombocytopenia is no longer specifically referred to in the US Food and Drug Administration manufacturer label; rather, there is a nonspecific reference to bleeding diathesis as a contraindication for thrombolysis. While no prospective or randomized controlled studies have been done to evaluate thrombocytopenia and the risk of hemorrhagic complications with tPA use, the expert consensus has set the threshold as platelet counts < 100,000/mm^3^ due to an expected increased risk of bleeding (Class III; Level of Evidence C) [1-3]. Few reports of patients with low platelet counts who were administered tPA are found in the literature as these individuals were excluded from the National Institute of Neurological Disorders and Stroke (NINDS) tPA trials [3-4]. Of the 21 thrombocytopenic patients treated with tPA found in pooled trial data, only one incident of intracerebral hemorrhage (ICH) was reported [3].

Although not an absolute contraindication to thrombolysis treatment, advanced age is a relative contraindication and an independent risk factor for poor outcomes irrespective of tPA use. The Stroke Prognostication using Age and National Institutes of Health Stroke Scale (SPAN; NIHSS) index was created by adding the patient’s age in years to their NIHSS score on presentation with acute ischemic stroke. A SPAN greater than or equal to 100 indicates a positive score and a high risk for ICH: a 42% risk of ICH in a SPAN-100-positive patient receiving tPA and a 19% risk in a SPAN-100-positive patient not receiving tPA [5]. Despite having an overall worse outcome, SPAN-100-positive patients treated with tPA had a significant reduction in the risk of severe disability or death compared to those without tPA and thus did benefit from intravenous (IV) thrombolysis [6]. This same reduction in risk of severe disability and death was not seen in SPAN-100-negative patients treated with or without tPA. Despite this evidence, many clinicians remain wary of administering tPA to a SPAN-100-positive patient likely secondary to the fear of ICH.

Here, we report a SPAN-100-positive, 90-year-old male patient with thrombocytopenia who presented with an acute ischemic stroke. The patient was treated with IV thrombolysis with rapid and significant improvement in stroke burden and no morbidity associated with the tPA.

## Case presentation

A 90-year-old male patient presented to the emergency department with left-sided weakness, facial droop, and slurred speech with onset two hours prior. He had a history of hypertension, dyslipidemia, and coronary artery disease and was status-post coronary artery bypass grafting in 1997 with no prior history of stroke. At baseline, the patient lived independently, was physically active, ambulated freely, and served as the primary caregiver for his wife who has Parkinson’s disease. The day prior to presentation, he had been chopping wood.

On admission, his blood pressure was 125/76, pulse was 60 beats/min, and his temperature was 95.9°F. The patient was awake and oriented but had significant dysarthria and unilateral upper motor neuron left facial weakness. He had a significant right gaze preference and left homonymous hemianopia. The left upper extremity was completely flaccid, and the left lower extremity had trace movement. He had a positive upgoing Babinski sign on the left. The patient’s NIHSS was 15. Non-contrast computed tomography (CT) imaging of his head showed no acute pathology, while a CT angiogram (CTA) of his head and neck showed bilateral carotid disease that was worse on the right but not flow-limiting. The CTA impression also indicated intracranial atherosclerosis, an open M1 branch on the right, and a possible partial occlusion or stenosis of his right superior M2 branch. Endovascular therapy would have taken at least another hour as the patient would have needed to be transferred to a different facility.

The patient demonstrated no signs or symptoms and had no clinical reason to be mildly thrombocytopenic. As a standard tPA bolus (10% of the total dose, based on 0.9 mg/kg dosing) was about to be administered, his platelet count was 85,000/mm^3^ with a prothrombin time of 15.1 seconds and an international normalized ratio of 1.27. Additionally, his SPAN-100 score was positive. His mild thrombocytopenia was technically an absolute contraindication for tPA. Nevertheless, given the patient’s quick hospital presentation and his high prehospitalization functional capacity, the decision was made with the patient’s family present that IV tPA be administered. Fibrinogen and serial clinical assessments were to be followed in case of hemorrhagic transformation with the potential for cryoprecipitate infusion. His baseline fibrinogen level at presentation was 333 mg/dL.

**Figure 1:**
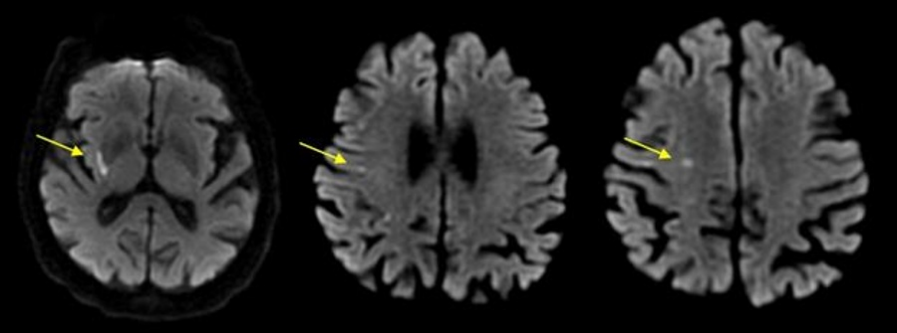
MRI Showing Ischemic Strokes Post-tPA Administration Yellow arrows: Diffusion-weighted magnetic resonance imaging demonstrating multiple acute scattered infarctions in the right middle cerebral artery distribution, including the right subinsular cortex (left image), right subcortical white matter (middle image), and right centrum semiovale (right image). MRI: magnetic resonance imaging; tPA: tissue plasminogen activator

Through the course of tPA infusion, the patient began regaining movement in his left upper and lower extremities. Roughly an hour after completion of the tPA administration, his strength on his left side was markedly improved. The patient’s motor strength was 4-/5 in the upper extremity compared to the plegia on initial presentation and 4-/5 in the lower extremity. Magnetic resonance imaging of the brain was consistent with acute scattered right middle cerebral artery ischemia (Figure 1). His fibrinogen level the following day (after tPA administration) was 255 mg/dL.

Over the nine days of hospitalization, the patient’s motor strength improved to 4+/5 in the left upper and lower extremities. He had mild dysphagia and required a modified diet on discharge. Of note, he developed mild confusion that worsened in the evenings with a questionable cognitive decline, both of which were suspected to be secondary to his stroke and superimposed delirium. His platelet count continued to remain below normal limits, with a nadir of 72,000/mm^3^ three days after tPA administration, before increasing to 81,000/mm^3^ prior to discharge. On his three-month follow-up evaluation, his platelet count remained mildly low at 85,000/mm^3^. Unfortunately, he did not follow-up with the stroke clinic.

## Discussion

Recently the AHA and ASA released a statement assessing the inclusion and exclusion criteria for recombinant tPA administration. Given the review of absolute and relative [1] contraindications to IV recombinant tPA for acute ischemic stroke by Fugate and Rabinstein and the AHA/ASA statement published, there are no large-scale prospective studies for the risk of hemorrhagic complications in patients with thrombocytopenia who receive IV tPA. There is also a lack of studies into the relationship between platelet count and risk of hemorrhagic complications with tPA [1].

Studies have indicated that prior to the administration of tPA in patients with acute ischemic stroke, it is not necessary to await platelet count results unless there is a suspicion for thrombocytopenia or bleeding diathesis due to the benefit of early thrombolysis outweighing the risk of inadvertent treatment of a thrombocytopenic patient [2-3, 7]. In Cucchiara’s study, only 0.3% of patients at the presentation of stroke had an unsuspected platelet count < 100,000/mm^3^ [7].

Notably, our patient’s thrombocytopenia was also in the setting of a positive SPAN-100 score. Due to the high risk of hemorrhagic transformation following tPA administration in this patient based on the SPAN-100 alone, a proper plan for reversal was important. This was accomplished by closely following his initial fibrinogen levels and the decrease in his fibrinogen level following thrombolysis. A decrease > 200 mg/dL six hours after treatment is associated with a greater ICH occurrence [8-9]. Cryoprecipitate has been used to attenuate the effects of tPA in clinically and radiographically evident ICH development, with fibrinogen levels guiding dosing and repeated administration [2, 8-9]. If in the setting of clinically and radiographically evident ICH development, cryoprecipitate has been used to attenuate the effects of tPA while monitoring fibrinogen levels to determine the need for further cryoprecipitate administration [2, 8-9].

Determining a more exact threshold at which the risk of hemorrhage exceeds the benefit from thrombolysis can provide clinicians with improved quantitative exclusion criteria for tPA use in patients with thrombocytopenia who present with acute ischemic stroke. Consideration should be made to consider moderate thrombocytopenia (i.e., 50,000 to 99,000/mm^3^) a relative contraindication to tPA rather than an absolute contraindication [10]. In a review evaluating bleeding risk in thrombocytopenic patients, Slichter concluded there was little correlation between the exact platelet count and bleeding risk, and in clinically stable patients, major bleeding was unusual in those with platelet counts > 50,000/mm^3^ [10].

## Conclusions

We often encounter cases with uncertainty regarding management, where high-quality data are not available. In these cases, clinical decisions must be made on an individual basis. If our patient, a high-functioning nonagenarian and the sole caretaker of his debilitated wife, was to complete his initial stroke without intervention, both his and his wife’s lives would likely be devastated. In all older patients with acute ischemic stroke presenting within the thrombolytic time-window, it is essential to discuss with the patient and family what the patient values the most: minimizing risk of mortality vs improving the odds of retaining functional independence and a high quality of life because optimizing the latter will likely come at the expense of the former. The risk of disability and death is higher with or without treatment, but thrombolysis will give the patient better odds towards improvement and functional independence. Continued evaluation of tPA eligibility criteria can improve the safety and increase the number of patients who can receive and benefit from thrombolysis treatment.
